# Associated factors and quality of life in women with urinary incontinence in southern Peru, 2023

**DOI:** 10.3389/fpubh.2024.1487330

**Published:** 2024-12-18

**Authors:** Gema Sologuren-García, Carmen L. Linares, Jackeline R. Flores, Gloria Escobar-Bermejo, Soledad Sotelo-Gonzales, Cristhel K. Fagerstrom

**Affiliations:** ^1^Jorge Basadre Grohmann National University, Tacna, Peru; ^2^School of Obstetrics and Childcare, Faculty of Medicine, Universidad Andrés Bello, Santiago, Chile

**Keywords:** pelvic floor, prevalence, quality of life, urinary incontinence, women

## Abstract

**Background:**

Urinary incontinence (UI), which can be classified as stress, urgency, or mixed, represents a public health problem that mainly affects adult women. This study aimed to determine the prevalence, association of sociodemographic and obstetric factors with the types of UI as well as the perceived impact on quality of life of women attending primary health facilities in the Tacna region from Peru.

**Methods:**

A quantitative, non-experimental, correlational, and cross-sectional study was conducted. Stratified sample. A total of 346 women aged 30 to 64 years were surveyed, applying two short version instruments: the Urinary Discomfort Inventory Questionnaire (UDI-6) and the Urinary Incontinence Impact Questionnaire (IIQ-7). Descriptive statistics, parameters of the multinomial logistic regression, with the *B* values, odds ratio (OR) and significance level (*p* < 0.05).

**Results:**

The prevalence of UI was 80.9%, with mixed UI being more frequent (48.8%). A significant association was found between the sociodemographic factor of educational level and type of UI (*p* = 0.004). Obstetric factors: higher frequency of mixed urinary incontinence (MUI) in vaginal delivery (30.6%) and newborns weighing 2,500–3,999 grams (35.5%). Additionally, an association between quality of life and type of urinary incontinence was observed, with a greater impact on those who had MUI (18.2%).

**Conclusion:**

There is a high prevalence of UI in women in the Tacna region, the sociodemographic associated is educational level. There is an association between quality of life and UI, with MUI being more frequent.

## 1 Introduction

The World Health Organization (WHO) recognizes UI as a major public health problem that affects millions of people worldwide, especially women (1). UI is not only a medical problem, it also has an impact on the psychological, social and economic aspects, due to the stigma and isolation it can cause, generating functional limitations and often negative relationships with people, couples and families (2–4).

The International Association of Urogynecology (IUGA) and the International Continence Society (ICS) have stablished standardized definitions of UI, considering it as an important medical issue that impacts quality of life. This regulatory framework facilitates the understanding and classification of UI, which is essential for its diagnosis, treatment and management. According to the ICS, UI is defined as “any involuntary loss of urine.” This definition includes different conditions, classified into types, according to the symptoms and circumstances that cause urine leakage (5), being more common in women (6). Among the types of UI are the urgency UI (UUI), effort UI (EUI) and mixed UI (MUI). EUI is characterized by involuntary urine leakage during activities that increase abdominal pressure, such as coughing, sneezing, or exercise; in contrast, UUI occurs along with an urgent need to urinate, while MUI has characteristics of both types (3, 5).

Furthermore, the ICS provides a clear framework regarding the prevalence of UI, highlighting its impact on public health and the importance of a comprehensive approach. The prevalence varies significantly according to the different studies and populations analyzed: in the United States, it was estimated that 61.8% of women experience some type of UI and more than 20% have moderate or severe UI (7). In Japan, the estimate for women between 20 and 64 years of age was 25.5% and increased with age (8). The percentage of women seeking help for the problem is low, 24% for Asia and 33% for Europe (9). Chile, Colombia, Mexico, and Costa Rica have policies and research that address their high prevalence and impact on quality of life (10–13). Finally, in southern Peru (14), a prevalence of 73.9% was found in pelvic floor dysfunction (PPD), with UI being the most frequent. UI tends to increase with age, being especially high in older women, due to factors such as aging, menopause and weakness in the pelvic floor musculature. This highlights the importance of well-designed epidemiological studies to better understand the magnitude of this condition and its effects on the affected population, especially since UI not only has physical implications, but also generates significant psychological and social problems (7).

The context of the study corresponds to the southern region of Peru (Tacna). This region presents geographical, sociodemographic, and healthcare access characteristics that affect the quality of life, especially among women, with diverse socioeconomic levels and a lack of specialized pelvic health care, which constitutes one of the main barriers. Additionally, 59.7% of the population are migrants, mainly from the Puno region (a high Andean area) (15), which has specific cultural characteristics, such as the presence of patriarchal families where women tend not to express their sexual health issues, as it is considered a taboo subject. These cultural dynamics perpetuate the lack of awareness and invisibility of health problems such as pelvic floor dysfunctions, which often remain unaddressed and untreated.

The main risk factors associated with UI in women include age (6, 7, 16), pregnancy and parity (14, 17, 18), history of hysterectomy, obesity, pelvic radiation, as well as pelvic floor affectation during childbirth (19, 20). Of these factors, parity is one of the most important, as childbirth can lead to pelvic muscle atrophy and nerve deterioration, increasing the risk of UI (3, 20). Weak connective tissue and high fetal weight at birth are important risk factors (21). In addition, being overweight has also been observed to be a risk factor for UI in women (14, 16), which highlights the importance of maintaining a healthy weight to prevent this condition, especially in young and middle-aged women (22). Strenuous exercise has been studied in recent years, and has been found to be a risk factor for UI, specially in women due to increased of intra-abdominal pressure and potential weakening of the pelvic floor (23). However, moderate physical activity and proper pelvic floor training can offer significant benefits and help prevent it. Pelvic floor muscle training (PFMT) is considered as the first choice treatment for women with UI, focusing on increasing strength and correcting their activation patterns (24). Recent evidence has demonstrated its effectiveness in the treatment of UI and other disorders (25).

The ICS has recommended the inclusion of a quality of life questionnaire in all UI-related studies, highlighting the importance of assessing the impact on individuals’ physical, psychological, and social well-being (26). Therefore, the adaptation of questionnaires and the validation of assessment tools in different populations, such as in Chile, reflect efforts to understand and address UI as a public health problem (11, 27). In addition, WHO’s recommendation to study and analyze the evolution of health systems, as demonstrated in the context of Peru, reflects a broader global perspective to address public health challenges, including UI (28).

The recognition and understanding of risk factors are essential for the design of strategies for the prevention, diagnosis and treatment of UI in women, as well as to promote pelvic floor health and improve quality of life in those affected. Therefore, the objective of the study was to determine the prevalence, association of sociodemographic and obstetric factors with the type of UI and to know the perception of its impact on the quality of life of women who attend first-level health facilities in the Tacna region—Peru.

## 2 Methodology

This study, with a quantitative approach, utilized a non-experimental and correlational cross-sectional design. The population consisted of 70,924 women aged 30 to 64 years, assigned to different healthcare networks of the Ministry of Health, from which a representative sample of 346 was selected. Each healthcare network was considered a stratum, and the following parameters were applied for sample calculation: population = 70,924, *z* = 1.96 (value of the normal distribution for 95% confidence), *p* = 0.5; *q* = 0.5, and a margin of error of 5.26%. Participants were randomly selected from each healthcare network. All women who agreed to participate in the study were included, regardless of their parity, without limitations in relation to the practice of sports and who signed the informed consent form. Those who presented psychic alterations or cognitive impairment were excluded. Below is the table detailing the study’s population and sample.

**Table:** 

Network	Population	Sample
Metropolitano	20,443	100
Cono Sur	26,310	128
Cono Norte	12,934	63
Litoral	2,443	12
Jorge Basadre	2,457	12
Frontera	3,656	18
Tarata	1,114	6
Candarave	1,083	5
Alto Andino	484	2
Total	70,924	346

Network dataset.

Ethical approval for the study was obtained from the Institutional Ethics and Research Committee of the Hipólito Unanue Hospital in Tacna, with the assigned code: 28-CIÉI-HHUT-2024.

### 2.1 Technique, instrument and data processing

Two measurement scales were used in short version: Urinary Discomfort Inventory Questionnaire (UDI-6), to determine prevalence and type of incontinence (29, 30) and the Impact of Urinary Incontinence (IIQ-7) on quality of life (31).

To determine the type of incontinence, specific questions from the UDI-6 questionnaire were used. Item 2 identified UUI, characterized by urinary leakage associated with a sudden and intense need to urinate. On the other hand, item 3 focused on EUI, which occurs when there is urinary leakage during physical activities or when pressure is exerted on the abdomen, such as coughing, sneezing, laughing, or lifting objects. When both items showed positive results, the person was considered to have MUI, which combines characteristics of the previous two types (30).

Various studies corroborate that both questionnaires have proven to be psychometrically valid and reliable, documenting key aspects such as validity, reliability and sensibility to change (29, 32, 33). Turning them in valuable tools in clinical practice and research. They were adapted to their short versions in 2005 and validated into other languages such as French, Swedish, Chinese, Arabic, Turkish and, Spanish in U.S. Spanish speakers (29, 34).

### 2.2 Procedure and statistical analysis

The analysis was executed using SSPS version 27 statistical software and Excel. To determine prevalence, type of UI and quality of life, descriptive statistics were used. For the association of variables, the parameters of the multinomial logistic regression, including the values of *B*, odds ratio (OR) and the significance level (*p* < 0.05) were determined.

## 3 Results

**Figure 1:**
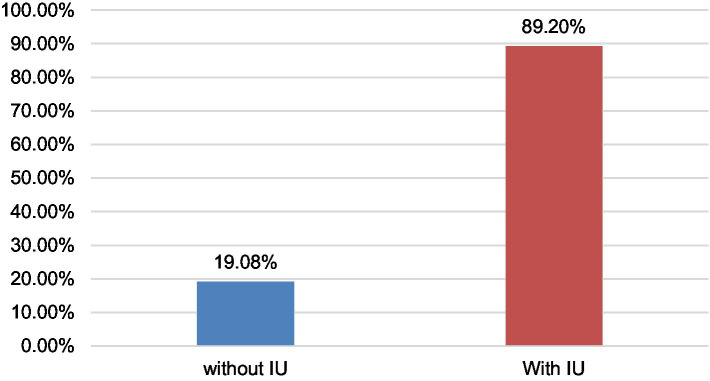
Prevalence of urinary incontinence in women in the Tacna region—2023.

**Figure 2:**
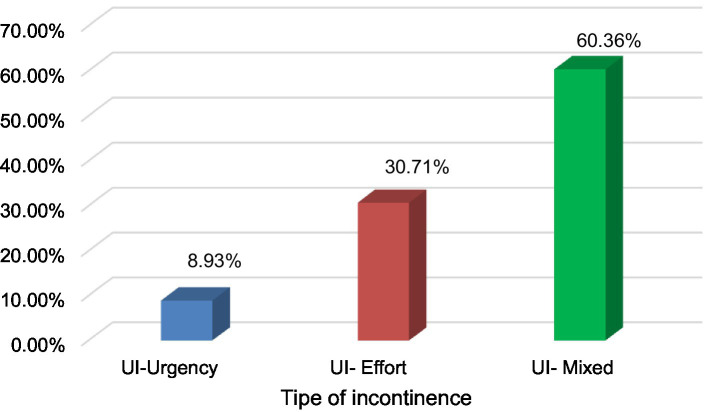
Types of urinary incontinence in women in the Tacna region—2023.

The prevalence of UI in women aged 30 to 64 years was 80.9% (Figure 1), with MU being the most frequent with 60.4% (Figure 2). This occurs in the group of 30 to 49 years old (35.8%) with overweight (21.4%) and obesity (18.5%); with secondary (23.7%) and higher (14.2%) levels of education, cohabiting (25.4%) and separated (12.4%), living in urban areas (41.3%). Educational level shows a significant association with stress urinary incontinence (OR = 4.431) and a possible association with mixed incontinence (OR = 2.441). Age has a slight relationship with stress incontinence (OR = 1.031), but it is not conclusive for other types of incontinence. Other factors, such as nutritional status, occupation, marital status, and place of origin, were not significant in most cases (Table 1).

**Table 1:** Association of socioeconomic factors and types of urinary incontinence in women of the Tacna region—2023.

Feature	Category	Type of urinary incontinence					Logistic regression		
		Without UI	UI-urgency	UI-effort	UI-mixed		UI-urgency	UI-effort	UI-mixed
		*n* (%)	*n* (%)	*n* (%)	*n* (%)				
Age	From 30 to 49	48 (13.9)	18 (5.2)	55 (15.9)	124 (35.8)	B	0.004	0.031	0.017
	From 50 to 59	12 (3.5)	7 (2.0)	21 (6.1)	32 (9.2)	OR	1.004	1.031	1.017
	From 60 and over	6 (1.7)	0 (0.0)	10 (2.9)	13 (3.8)	p	0.860	0.680	0.270
Nutritional status	Normal	16 (4.6)	6 (1.7)	28 (8.1)	31 (9.0)	B	0.318	0.560	−0.279
	Overweight	25 (7.2)	11 (3.2)	33 (9.5)	74 (21.4)	OR	1.375	1.750	0.757
	Obese	25 (7.2)	8 (2.3)	25 (7.2)	64 (18.5)	p	0.558	0.185	0.472
Level of education	No level	1 (0.3)	1 (0.3)	1 (0.3)	5 (1.4)	B	0.664	1.489	0.893
	Primary	8 (2.3)	1 (0.3)	22 (6.4)	33 (9.5)	OR	1.942	4.431	2.441
	High school	28 (8.1)	15 (4.3)	45 (13.0)	82 (23.7)	p	0.195	0.004	0.052
	Superior	29 (8.4)	8 (2.3)	18 (5.2)	49 (14.2)				
Occupation	Housewife	25 (7.2)	13 (3.8)	40 (11.6)	70 (20.2)	B	0.388	0.470	0.225
	Independent	24 (6.9)	6 (1.7)	29 (8.4)	61 (17.6)	OR	1.473	1.600	1.253
	Dependent	17 (4.9)	6 (1.7)	17 (4.9)	38 (11.0)	p	0.598	0.271	0.546
Marital status	Married woman	14 (4.0)	0 (0.0)	16 (4.6)	24 (6.9)	B	0.485	0.152	−0.131
	Cohabitant	29 (8.4)	13 (3.8)	44 (12.7)	88 (25.4)	OR	1.625	1.164	0.877
	Separate	13 (3.8)	8 (2.3)	19 (5.5)	43 (12.4)	p	0.514	0.781	0.831
	Single	10 (2.9)	4 (1.2)	7 (2.0)	12 (3.5)				
	Widow	0 (0.0)	0 (0.0)	0 (0.0)	2 (0.6)				
Origin	Urban	56 (16.2)	23 (6.6)	72 (20.8)	143 (41.3)	B	0.720	−0.085	−0.018
	Rural	10 (2.9)	2 (0.6)	14 (4.0)	26 (7.5)	OR	2.054	0.918	0.982
						p	0.376	0.850	0.964

Source: Database based on the survey applied to women in Tacna. The reference category is without UI.

Regarding habits, alcohol consumption shows a significant negative association with mixed MUI (*B* = −0.730, OR = 0.482, *p* = 0.043) and with UUI (*B* = −0.832, OR = 0.435, *p* = 0.010). However, for SUI (*B* = −0.830, OR = 0.436, *p* = 0.137), no significant association was found. Coffee consumption (*B* = 0.679, OR = 1.971, *p* = 0.188) appears to be related to an increased likelihood of MUI, but this association is not significant. Although for UUI (*B* = 1.242, OR = 3.462, *p* = 0.214) the coefficient suggests a possible positive association. However, for EUI (*B* = 1.989, OR = 7.308, *p* = 0.049), this relationship is not significant (Table 2).

**Table 2:** Association of habits and types of urinary incontinence in women of the Tacna region—2023.

Feature	Category	Type of urinary incontinence					Logistic regression		
		Without UI	UI-urgency	UI-effort	UI-mixed		UI-urgency	UI-effort	UI-mixed
		*n* (%)	*n* (%)	*n* (%)	*n* (%)				
Physical exertion	No	47 (13.6)	16 (4.6)	66 (19.1)	97 (28.0)	B	−0.330	0.288	−0.608
						OR	0.719	1.334	0.545
	Yes	19 (5.5)	9 (2.6)	20 (5.8)	72 (20.8)	p	0.660	0.308	0.103
Chronic cough	No	63 (18.2)	25 (7.2)	79 (22.8)	157 (45.4)	B	−0.421	−0.621	−0.473
						OR	0.651	0.537	0.623
	Yes	3 (0.9)	0 (0.0)	7 (2.0)	12 (3.5)	p	0.520	0.293	0.621
Amount of coffee	Do not take	48 (13.9)	15 (4.3)	66 (19.1)	120 (34.7)	B	1.242	1.989	0.679
	1 cup/day (less than 200 mg)	13 (3.8)	9 (2.6)	19 (5.5)	41 (11.8)	OR	3.462	7.308	1.971
	2 to 4 cup/day (100 to 400 mg)	5 (1.4)	1 (0.3)	1 (0.3)	8 (2.3)	p	0.214	0.049	0.188
Alcohol	No	53 (15.3)	16 (4.6)	55 (15.9)	112 (32.4)	B	−0.830	−0.832	−0.730
						OR	0.436	0.435	0.482
	Yes	13 (3.8)	9 (2.6)	31 (9.0)	57 (16.5)	p	0.137	0.010	0.043

Source: Database based on the survey applied to women in Tacna. The reference category is without UI.

MUI predominated in women with a history of vaginal delivery (30.6%) and those with both cesarean and vaginal deliveries (7.8%). The mode of delivery (*B* = −1.270; OR = 0.281, *p* = 0.031) is significantly associated with a lower likelihood of having mixed urinary incontinence. However, for UUI (*B* = 1.163; OR = 3.200, *p* = 0.947), there is a trend towards increasing the likelihood, but it is not significant. Regarding SUI (*B* = −0.816; OR = 0.442, *p* = 0.240), the mode of delivery shows a trend towards reducing the likelihood, but this association is also not significant. A higher newborn weight tends to increase the likelihood of MUI (*B* = 0.565; OR = 1.760, *p* = 0.108), although this relationship is not significant (Table 3).

**Table 3:** Association of obstetric factors and types of urinary incontinence in women of the Tacna region—2023.

Feature	Category	Type of urinary incontinence					Logistic regression		
		Without UI	UI-urgency	UI-effort	UI-mixed		UI-urgency	UI-effort	UI-mixed
		*n* (%)	*n* (%)	*n* (%)	*n* (%)				
No. of births	None	11 (3.2)	4 (1.2)	8 (2.3)	12 (3.5)	B	0.556	−0.913	−1.268
	From 1 to 2	39 (11.3)	17 (4.9)	49 (14.2)	95 (27.5)	OR	1.744	0.401	0.282
	From 3 to more	16 (4.6)	4 (1.2)	29 (8.4)	62 (17.9)	p	0.538	0.209	0.056
Birth route	No delivery	11 (3.2)	4 (1.2)	8 (2.3)	12 (3.5)	B	1.163	−0.816	−1.270
	Caesarean section	19 (5.5)	4 (1.2)	14 (4.0)	24 (6.9)	OR	3.200	0.442	0.281
	Vaginal	30 (8.7)	16 (4.6)	54 (15.6)	106 (30.6)	p	0.947	0.240	0.031
	Cesarean and vaginal section	6 (1.7)	1 (0.3)	10 (2.9)	27 (7.8)				
Heaviest newborn	Without RN	11 (3.2)	4 (1.2)	8 (2.3)	12 (3.5)	B	0.090	−0.606	0.565
	From 2,500 to 3,999	37 (10.7)	21 (6.1)	54 (15.6)	123 (35.5)	OR	1.095	0.545	1.760
	From 4,000 to more	18 (5.2)	0 (0.0)	24 (6.9)	34 (9.8)	p	0.551	0.927	0.108

Source: Database based on the survey applied to women in Tacna. The reference category is without UI.

Likewise, it was found that women with urinary incontinence have a significantly higher likelihood of experiencing a mild impact on their quality of life (*B* = 2.658; OR = 14.264; *p* = 0.000), as well as a moderate impact (*B* = 2.188; OR = 8.915; *p* = 0.036) compared to those without urinary incontinence. This association is statistically significant (Table 4).

**Table 4:** Association of types of urinary incontinence and their impact on the quality of life of women of the Tacna region—2023.

Urinary incontinence	Impact on quality of life				Logistic Regression	
	No impact	Slight	Moderate		Slight	Moderate
	*n* (%)	*n* (%)	*n* (%)			
Without UI	63 (18.2)	2 (0.6)	1 (0.3)			
UI-urgency	22 (6.4)	3 (0.9)	0 (0.0)	B	2.658	2.188
UI-effort	69 (19.9)	14 (4.0)	3 (0.9)	OR	14.264	8.915
UI-mixed	106 (30.6)	48 (13.9)	15 (4.3)	p	0.000	0.036

Source: Database based on the survey applied to women in Tacna. The reference category is no impact.

## 4 Discussion

The study showed that out of every five women, four have UI, with MUI being more frequent; Milsom and Gyhagen (35), in a systematic review, argues that other studies have reported prevalences between 25 and 45% and that increase with age, exertional UI becoming more common. Llajaruna Zumaeta and Urbina Quispe (36) showed that this subtype of UI has a prevalence that varies between 40 and 50%. On the other hand, Khan et al. (37) in a study carried out in India found that the older the age, the more cases of MUI increase, unlike the present study where MUI occurs at younger ages (30 to 49 years). Salo et al. (38) found, using a logistic regression model, in a population from Finland, that MUI is associated with poor work capacity (OR 2.51, 95% CI 1.68–3.74), especially in young women. It should be noted that UI is a common condition among women. In this regard, Turk et al. (39) states that one in seven women suffer from some type of UI. Despite its high prevalence, about half of those with clinically significant UI are undiagnosed and untreated (40).

Regarding sociodemographic factors, the data suggest some trends, such as a higher frequency of MUI in women aged 30 to 49 years, with overweight and obesity, medium and high educational levels, housewives and self-employed women, cohabiting and separated, as well as coming from urban areas. Different authors found similar results where UI was be associated with factors such as age, obesity, menopause, rural area of residence, among others (37, 41, 42). Other research suggests that MUI it’s an underestimated problem (37, 43). Islam et al. (44), in a research conducted in Bangladesh identified that women in the highest wealth quintile (*p* = 0.009) and underweight women had a lower risk of emergency UI (*p* = 0.018), constituting protective factors. This research also found that educational level has a significant association with EUI, which may be due to the fact that women with higher educational levels tend to be more informed about health issues, are more likely to consult medical professionals when experiencing symptoms, and are more open to discussing these topics. Digging deeper into these findings could guide more targeted interventions based on the identified risk profiles.

MUI was more frequent in women with 1–2 deliveries, predominating in women with vaginal deliveries. Similar results were obtained in other studies where parity is an associated factor (6, 18, 45). Other researchers assert multiparity as a risk factor (3, 46). This condition, along with factors such as gestational age, history of miscarriages, and constipation, are significantly associated with UI during pregnancy (46–48). Physiological changes, such as increased abdominal pressure, progesterone levels, and pelvic floor injuries, can increase susceptibility to it (18, 48).

It was found that the mode of delivery (*B* = −1.270; OR = 0.281, *p* = 0.031) is significantly associated with a lower likelihood of developing MUI. This may be due to the fact that nearly a quarter of the sample had a cesarean delivery, which, by avoiding the birth canal, reduces the risk of damage to these structures.

UI is more common in multiparous women and can persist even after childbirth (7, 47). Therefore, it is crucial that health professionals recognize parity as a significant risk factor when evaluating and treating UI, especially during the prenatal and postpartum period.

Although no significant association was found with newborn weight, research suggested that vaginal delivery with higher birth weight infants may increase the risk of developing postpartum UI (21, 49). In addition, it has been observed that obstetric trauma during childbirth, particularly in cases of shoulder dystocia or instrumental births, could trigger it (45), determining that the weight of the newborn may be a relevant risk factor (50). Understanding this connection can be critical to improving its prevention, diagnosis, and treatment, providing comprehensive and personalized care to those who are affected by this condition. In this regard, the effectiveness of pelvic floor muscle training in the treatment of UI has been recognized, suggesting the potential for non-invasive interventions to address this issue (26). However, a study conducted in Japan found that less than 5% of individuals with UI had experience with pelvic floor muscle training and medical care (8). The literature also emphasizes the need for comprehensive care and support for individuals with UI, particularly among older adults, where physiotherapeutic treatment and pelvic floor muscle training can effectively improve muscle strength and quality of life (51, 52).

The findings suggest that UI has a significant negative impact on the quality of life of women in the Tacna region, who are approximately 14 times more likely to have their quality of life mildly affected and almost 9 times more likely to experience a moderate impact. This result coincides with the report by Moyolema Chicaiza (6), who points out that UI significantly affects the daily lives of women who suffer from it. The amount of urine leakage and frequency with which it occurs limit and impede personal, work, social and recreational activities (18, 48), and may even influence relationships, sexuality and general well-being (53). This observation coincides with previous research that has assessed quality of life in women with UI using standardized and validated questionnaires. For example, the “King’s Health Questionnaire,” adapted and validated for Portuguese women with UI, has shown how this condition negatively affects quality of life, reflecting similar limitations in social participation and general well-being (54). In addition, studies on strengthening the pelvic floor muscle have evidenced improvements in both quality of life and self-perception of women, highlighting the relevance of specific interventions in these cases (55). These results support and extend the understanding of the impact of UI on various aspects of daily life, aligning with the findings of this study.

Despite the negative impact of UI on quality of life, many women are reluctant to seek medical care for this condition (42, 56). The lack of open communication about UI is a clear issue, as a significant portion of those affected do not mention the condition, and only a minority seek professional care (9). Among the main reasons for not seeking medical advice are the embarrassment of discussing the issue, especially with male doctors, and the mistaken belief that it is a common or incurable condition. This reluctance is a complex phenomenon influenced by various factors (57). In this regard, Vallana Sala (58) mentions that it is not only due to cultural differences, but also to previous experiences with medicine, considering that the quality of medical care plays a crucial role in women’s willingness to seek treatment (59). The studies maintain that women with a lower level of education are at greater risk of not seeking medical attention. On the other hand, it has been observed that most of those affected do not seek medical attention, either because they consider it not to be serious or because they see it as a normal aging process, which contributes to normalizing UI (14, 60). Improving education about UI, treatment options, and promoting lifestyle changes offer significant opportunities (37). Consequently, the impact on women’s quality of life has been a focal point in several studies, emphasizing the need for comprehensive care and support for those affected (61). Despite its high prevalence, the discomfort caused by this condition is often accepted as normal by the general population, highlighting the urgent need to increase education on the subject (14).

UI can present itself in different forms (effort, urgency or mixed), mainly affecting women and older adults. A comprehensive approach, addressing both physical, emotional, and social aspects is essential to improve the quality of life of those affected.

Given that information about prior treatment was not included, there is a possibility that the results are underestimating or overestimating the true prevalence and its associated factors. This limitation should be considered when interpreting the findings, as women who received treatment might be less likely to present symptoms at the time of the study. In future studies, it would be important to collect data on UI treatment to adjust the analyses and account for this potentially confounding variable. It is recommended to continue researching and developing effective strategies to diagnose, treat and manage UI in women, in order to promote general well-being and health.

## 5 Conclusion

This study reveals a high prevalence of UI (80.9%), the most common form was MUI (48.8%). Regarding sociodemographic factors, an association was found with the level of education, obstetric risk factors were not associated with the types of UI: route of delivery, history of vaginal delivery, and newborn weight. Finally, an association was found between negative impact on quality of life and UI.

## Data Availability

The original contributions presented in the study are included in the article/supplementary material, further inquiries can be directed to the corresponding author.
